# Supplementary material for: Label-free imaging of immune cell dynamics in the living retina using adaptive optics
Source: eLife. 2020 Oct 14;9:e60547. doi: 10.7554/eLife.60547 (PMC7556865; doi:10.7554/eLife.60547)
Supplement: Transparent reporting form [file elife-60547-transrepform.docx]

***eLife’s* transparent reporting form**

We encourage authors to provide detailed information *within their submission* to facilitate the interpretation and replication of experiments. Authors can upload supporting documentation to indicate the use of appropriate reporting guidelines for health-related research (see [EQUATOR Network](http://www.equator-network.org/%20)), life science research (see the [BioSharing Information Resource](https://biosharing.org/)), or the [ARRIVE guidelines](http://www.plosbiology.org/article/info:doi/10.1371/journal.pbio.1000412) for reporting work involving animal research. Where applicable, authors should refer to any relevant reporting standards documents in this form.

If you have any questions, please consult our Journal Policies and/or contact us: [editorial@elifesciences.org](mailto:editorial@elifesciences.org).

**Sample-size estimation**

- You should state whether an appropriate sample size was computed when the study was being designed
- You should state the statistical method of sample size computation and any required assumptions
- If no explicit power analysis was used, you should describe how you decided what sample (replicate) size (number) to use

Please outline where this information can be found within the submission (e.g., sections or figure legends), or explain why this information doesn’t apply to your submission:

This information can be found in the following manuscript sections:

Type and Number of mice: Methods – Mouse strains, Cell migration measurement and Blood flow measurement

Number and types of vessels imaged: Methods – Blood flow measurement

Unless otherwise described, data is reported as population standard deviation

relative to mean. Where compared across mice, standard deviation represents variability across the group (e.g. vessels in different mice of the same vessel

type). We did not use power analysis to determine sample size.

**Replicates**

- You should report how often each experiment was performed
- You should include a definition of biological versus technical replication
- The data obtained should be provided and sufficient information should be provided to indicate the number of independent biological and/or technical replicates
- If you encountered any outliers, you should describe how these were handled
- Criteria for exclusion/inclusion of data should be clearly stated
- High-throughput sequence data should be uploaded before submission, with a private link for reviewers provided (these are available from both GEO and ArrayExpress)

Please outline where this information can be found within the submission (e.g., sections or figure legends), or explain why this information doesn’t apply to your submission:

Biological replication was investigated in several ways:

1) By examining cell motility, red blood cell velocity and vessel lumen diameter across six mice (addressed in Figure 2).

2) For blood flow measurement, thousands of repeat measures were performed sequentially in the same vessel for 1 second allowing serial measures of flow variability as a function of cardiac cycle (Figure 2). We observe similar but not identical velocity measures as a function of each heartbeat. This reveals subtle differences in true biological replication of blood velocity.

Technical replicates were conducted on the blood velocity software in two ways:

1) for blood velocity, the Radon code revealed the same velocity profile when run on the same data set multiple times (data not shown)

2) the code solved the correct local angle as confirmed with a ground-truth data set where local angles were known a priori.

For blood flow, algorithmically, conditions of velocity determination were identified in methods which provided limits on velocity bandwidth based on spatio-temporal resolution and angle search space.

Number of experiments performed, mice imaged and outlier handling:

For Figure 1, representative images from cohort of eleven (panels A to D, J) and three C57BL/6J mice (E), three CD68-GFP mice (F and H) and four Cx3cr1-GFP mice (G and I) are shown. For Figure 2, data from six mice is shown, for population analysis of immune cell motility and blood flow. Mice/imaging sessions with insufficient image quality due to ocular media opacities were not included, as determined by expert users. Quantitatively, reversible severe vitritis or cataract precluding AOSLO imaging occurred at a peak inflammation timepoint in 25% of the eyes induced with EIU in this work. Overall, numbers types, sex and ages of mice are summarized in the table below.

| **Mouse Strain** | **Number of mice** | **Gender** | **Age** |
| --- | --- | --- | --- |
| C57BL/6J for qualitative assessment | 3 (from starting group of 5) | Male | 6-12 weeks |
| C57BL/6J for blood flow and cell motility timecourse | 6 (from starting group of 7) | Male | 6-12 weeks |
| C57BL/6J for Ly6G antibody labelling | 3 | Male | 6-12 weeks |
| CD68-GFP | 3 | Male | 6-12 weeks |
| Cx3CR1-GFP | 4 | Male | 6-8 months |

**Statistical reporting**

- Statistical analysis methods should be described and justified
- Raw data should be presented in figures whenever informative to do so (typically when N per group is less than 10)
- For each experiment, you should identify the statistical tests used, exact values of N, definitions of center, methods of multiple test correction, and dispersion and precision measures (e.g., mean, median, SD, SEM, confidence intervals; and, for the major substantive results, a measure of effect size (e.g., Pearson's r, Cohen's d)
- Report exact p-values wherever possible alongside the summary statistics and 95% confidence intervals. These should be reported for all key questions and not only when the p-value is less than 0.05.

Please outline where this information can be found within the submission (e.g., sections or figure legends), or explain why this information doesn’t apply to your submission:

This information can be found in the methods section where number of samples, mice,

and repeat measures are described. Specific sections: Methods – Statistical analysis, Mouse strains, Cell migration measurement, Blood flow measurement.

(For large datasets, or papers with a very large number of statistical tests, you may upload a single table file with tests, Ns, etc., with reference to sections in the manuscript.)

**Group allocation**

- Indicate how samples were allocated into experimental groups (in the case of clinical studies, please specify allocation to treatment method); if randomization was used, please also state if restricted randomization was applied
- Indicate if masking was used during group allocation, data collection and/or data analysis

Please outline where this information can be found within the submission (e.g., sections or figure legends), or explain why this information doesn’t apply to your submission:

This manuscript uses automated computer algorithms. The blood flow algorithm is

agnostic to the vessel lumen diameter therefore it cannot introduce subjective bias in

blood velocity determination. This is a benefit from human subjective measurements which may introduce bias and has been previously reported. As per above, masking was not performed. All data was used without rejection of vessel outliers for blood flow analysis. Vessels were grouped based on their diameter and type (arteriole/venule). Diameter was measured objectively. Vessel type was indicated by direction of flow (arteriole: from the disc, venule: to the disc.)

**Additional data files (“source data”)**

- We encourage you to upload relevant additional data files, such as numerical data that are represented as a graph in a figure, or as a summary table
- Where provided, these should be in the most useful format, and they can be uploaded as “Source data” files linked to a main figure or table
- Include model definition files including the full list of parameters used
- Include code used for data analysis (e.g., R, MatLab)
- Avoid stating that data files are “available upon request”

Please indicate the figures or tables for which source data files have been provided:

-Matlab code is provided (https://github.com/abyjoseph1991/single_cell_blood_flow)

-Global and local variables are contained within above code

-Raw AOSLO data is large in size, constituting videos adding upto terabytes of data. Representative videos are provided with the manuscript as rich media files where possible. Data from our recent eLife paper (Joseph et al. 2019), upon which our current Research Advance submission is based, is available on Zenodo (https://doi.org/10.5281/zenodo.2658767).
